# 2140 Clinical and translational research (CTR) platform for undergraduate health sciences programs (UHSP) at University of Puerto Rico-Medical Sciences Campus (UPR-MSC) and Universidad Central del Caribe (UCC): Pipeline for students and faculty

**DOI:** 10.1017/cts.2018.199

**Published:** 2018-11-21

**Authors:** Rubén G. García, Margarita Irizarry-Ramírez, Efraín F. Rivera, Carlamarie Noboa, José Moscoso-Álvarez, María E. González-Méndez, Mildred I. R. Vázquez

**Affiliations:** 1 University of Puerto Rico-Medical Sciences Campus; 2 Title V Cooperative MSC-UCC

## Abstract

OBJECTIVES/SPECIFIC AIMS: The University of Puerto Rico-Medical Sciences Campus and Universidad Central del Caribe, through the Title V Cooperative Project, devised a clinical and translational research (CTR) platform to pipeline students/faculty of undergraduate health sciences programs into CTR. Educational interventions in CTR—introductory intervention (II) and Annual Symposium (AS)—were designed to promote awareness, stimulate interest of students and faculty in CTR. METHODS/STUDY POPULATION: In the II the participants (n=159) were surveyed before and after a presentation and panel discussion about CTR. In addition, after the sessions—plenary, panel, and workshop—about CTR, the participants of AS (n=42) were surveyed for satisfaction and learning experience in CTR. RESULTS/ANTICIPATED RESULTS: Most participants of the II, 134 (84.3%) were students. In total, 58 (58, 36.5%) completed the post II survey. Of these, 53.4% satisfactorily defined the CTR concept Versus only 31.0% that could define CTR in the pre survey, 47 (81.7%) were unable to identify a CTR researcher and 45 (78.3 %) expressed interest in learning about CTR. In total, 28 (28, 66.7%) participants of the AS completed the satisfaction survey, out of which 17 (60.6%) were students. One hundred percent (100%) agreed that the AS served as a vehicle to increase their knowledge in CTR. DISCUSSION/SIGNIFICANCE OF IMPACT: The educational interventions demonstrated to be an effective strategy to promote awareness and stimulate interest of students and faculty in CTR. In addition, the results obtained, provided valuable baseline information for the planning—development of training cycles in CTR.

